# Projection mapping system for laser speckle contrast image: feasibility study for clinical application

**DOI:** 10.1117/1.JBO.28.9.096001

**Published:** 2023-09-04

**Authors:** Subin Park, Insun Yeum, Donghwan Ko, Byungjo Jung

**Affiliations:** Yonsei University, Department of Biomedical Engineering, Wonju-Si, Republic of Korea

**Keywords:** laser speckle contrast image, projection mapping, blood flow monitoring, projector

## Abstract

**Significance:**

Laser speckle contrast images (LSCIs) have been utilized to monitor blood flow perfusion. However, they have conventionally been observed on monitor screens, resulting in potential spatial mismatching between the imaging region of interest (IROI) and monitor screen.

**Aim:**

This study proposes a projection mapping (PM) system for LSCIs (PMS_LSCI) that projects LSCIs to directly observe the blood flow perfusion in the IROI.

**Approach:**

The PMS_LSCI consists of a camera, imaging optics, a laser projector, and graphic user interface software. The spatial matching in the regions of interest was performed by adjusting the software screen of the LSCI in the IROI and evaluated by conducting *in-vitro* and *in-vivo* studies. An additional *in-vivo* study was performed to investigate the feasibility of real-time PM of the LSCI.

**Results:**

The spatial mismatching in the regions of interest was ranged from 2.74% to 6.47% depending on the surface curvature. The PMS_LSCI could enable real-time PM of LSCI at four different blood flow states depending on blood pressure.

**Conclusions:**

The PMS_LSCI projects the LSCI in the IROI by interacting with a projector instead of the monitor screen. The PMS_LSCI presented clinical feasibility in the *in-vitro* and *in-vivo* studies.

## 1 Introduction

Laser speckle contrast imaging is a non-invasive imaging technique widely employed in biomedical studies to evaluate and monitor blood flow perfusion. It utilizes the time variation of laser speckle patterns, which are randomly varying interference patterns caused by the scattering of moving particles (e.g., red blood cells in the vasculature). The relative velocity of moving particles can be obtained by calculating the speckle index, which indicates the contrast variation in the laser speckle contrast image (LSCI).^1^ The LSCI can be obtained by utilizing the spatial mode algorithm, which is useful for monitoring blood flow perfusion as a function of time, or the temporal mode algorithm, which is useful for comparing blood flow perfusion before and after an event. The spatial mode algorithm is based on moving averaging of N×N window pixels in an image, which determines the spatial resolution and speckle contrast. In general, 5×5 or 7×7 windows are employed; however, the averaging window size depends on the hardware configuration, speckle contrast, and spatial resolution.^2^^,^^3^ The temporal mode algorithm is based on pixel averaging at the same pixel location after acquiring multiple images and provides higher spatial resolution than the spatial mode algorithm.^4^ The ability to obtain LSCIs in real-time depends on the speed of image acquisition and processing, as well as the computer performance.

LSCIs have been widely used in various clinical applications, such as diagnosis of keloids classification,^5^^–^^9^ burn evaluation,^10^^–^^13^ observation of microcirculation following treatment,^14^^–^^17^ robot-assisted surgery,^18^ intraoperative cerebral blood flow monitoring,^19^^–^^21^ surgery monitoring,^22^^–^^25^ reperfusion evaluation after tissue transplantation,^26^^,^^27^ prediction of tissue necrosis risk,^28^^–^^30^ and reperfusion evaluation after oral wound healing.^31^^,^^32^

To ensure accurate measurement in the region of interest, the imaging region of interest (IROI) must be matched with the LSCI displayed on the monitor screen, which may be time-consuming due to potential spatial mismatching between the IROI and the monitor screen. To address the issue, previous studies have tried aligning the LSCI with a white light image on a monitor screen^19^^,^^33^ or using a projector to overlay an LSCI on a surgical microscope.^34^ Another promising study involved near-infrared subcutaneous vein imaging based on a projection mapping (PM) that selectively extracted the vein morphology and projected it onto the IROI to observe the vein morphology in the IROI directly.^35^

This study proposes a PM system for LSCIs (PMS_LSCI) that projects LSCIs to observe the blood flow perfusion in the IROI directly, eliminating the requirement of the monitor screen. The spatial mismatching between the IROI and projection region of interest (PROI) was quantitatively evaluated by conducting *in-vitro* vascular optical tissue phantom and *in-vivo* human studies. Graphic user interface (GUI) software was developed using MATLAB (MATLAB, MathWorks) for image acquisition and processing in the PMS_LSCI.

## 2 Materials and Methods

### 2.1 Development of PMS_LSCI

**Fig. 1:**
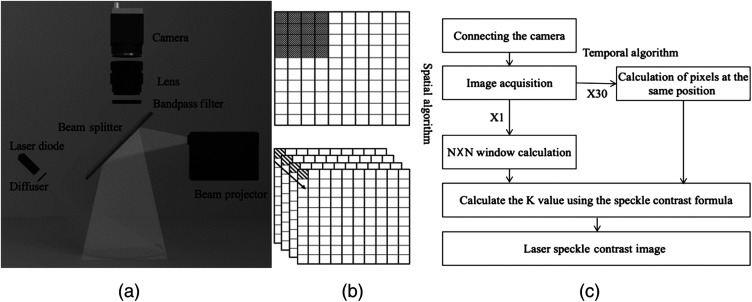
(a) Schematic of the projection imaging system for LSCI, (b) computation window of LSCI in spatial (upper image) mode based on moving averaging of N×N window pixels in an image and temporal (lower image) mode based on pixel-by-pixel averaging at the same pixel location in multiple images, and (c) flowchart of the GUI software used to compute LSCI in spatial or temporal mode.

Figure 1(a) shows the schematic illustration of the PMS_LSCI consisting of a 785 nm diode laser (L785P090, Thorlabs), optical diffuser (#35-869, Edmund Optics), monochrome camera (DCC3240N, Thorlabs), optical imaging lens (#67-714, Edmund Optics), bandpass filter (#65-723, Edmunds Optics) in front of the lens, laser projector (MP-CL1, Canon, Japan), and beam splitter (#62-882, Edmunds Optics) aligned at 45 deg.

To ensure clear imaging, the PMS_LSCI utilized a fixed working distance of 15 cm with a field of view of 23.9 deg, providing an IROI area of 9.8  cm×7  cm, and did not use other working distances that could result in image blurring. The laser projector with 1920×720  pixel resolution was placed perpendicular to the camera by using the beam splitter to project the LSCI onto the IROI. A diffuse reflectance target (SRT-99-100, Labsphere Inc.) with 99% reflectance was placed at the working distance to evaluate the laser distribution in the IROI. The evaluation was performed by calculating the coefficient of variation, which is the ratio of the standard deviation to the mean. The laser speckle image was acquired from the diffuse reflectance target in a dark room and column-averaged to obtain the two-dimensional (2D) beam profile for calculating the coefficient of variation.

MATLAB was used to develop GUI software that mainly acquires laser speckle images and computes LSCIs in either spatial or temporal mode. In this study, the LSCI in spatial mode was used for potential real-time PM in the IROI because this mode is faster than the temporal mode. The computer was equipped with a CPU (i7-1075H, Intel), 32 GB of memory, and a graphics processing unit (GTX 1650Ti, NVIDIA). Figures 1(b) and 1(c) show the schematics of the computation window of LSCI in spatial (upper image) and temporal (lower image) modes and the flowchart of the GUI software to compute the LSCI, respectively.

### 2.2 Evaluation of Spatial Matching in the Regions of Interest

The spatial matching between the IROI and PROI is very important in PMS_LSCI. Although the LSCI can be obtained without markers and projected, ensuring spatial matching between the IROI and PROI may be difficult. The LSCI must be precisely projected in the identical IROI to avoid blood perfusion artifacts due to spatial mismatching in the regions of interest (SMROI). White light images were obtained from a right lower arm and hand with square and circular markers to mask the exact shapes and positions in PM accurately. These images were projected onto the IROI by manually adjusting the position of the GUI screen to match the markers of the PROI spatially to those of the IROI. The SMROI was evaluated by calculating the number of pixels mismatched in the displacement of markers between the IROI and PROI. This evaluation was also performed in both *in-vitro* and *in-vivo* studies using the LSCI instead of white light images.

### 2.3 *In-Vitro* Evaluation of PMS_LSCI

An *in-vitro* evaluation of the PMS_LSCI was performed using a vascular optical tissue phantom made from room temperature-vulcanizing silicone with optical tissue properties at 785 nm. A square marker was attached to the vascular optical tissue phantom to investigate the SMROI. A milk solution was circulated through the vascular optical tissue phantom by a peristaltic pump (PP-150D, PLTECH, Korea) to simulate blood flow. LSCIs were obtained at various revolutions per minute (RPM) (60, 80, 100, and 120) and projected onto the vascular optical tissue phantom as a function of RPM. The PM of the LSCI was imaged using a smartphone camera that was closely placed to the beam splitter. To quantify the LSCI, the blood flow index was calculated as $1/K^{2}$.^36^ The statistical analysis of the relationship between blood flow index and RPM was performed using IBM SPSS software (SPSS 22.0, IBM). A paired-sample t-test was used to compare the blood flow index between the two groups, and statistical significance was determined if P<0.05.

### 2.4 *In-Vivo* Evaluation of PMS_LSCI

To investigate the SMROI due to surface curvature, LSCIs were obtained by attaching square markers on various parts of the hand, such as the volar wrist, fingers, and flat and curved dorsal hands. The SMROI was evaluated by calculating the displacement of the markers between the IROI and PROI. A sphygmomanometer cuff was applied to the right upper arm to control blood flow and evaluate changes in blood perfusion in the hand. The LSCIs were acquired and projected onto the IROI at four different states: normal without occlusion, occlusion by tightening the cuff, reperfusion by releasing the cuff, and returning to normal 2 min after releasing the cuff. The *in-vivo* experiment was approved by the Bioethics Committee of Yonsei University (1041849-202111-BM-192-01).

## 3 Results

### 3.1 Development of PMS_LSCI

**Fig. 2:**
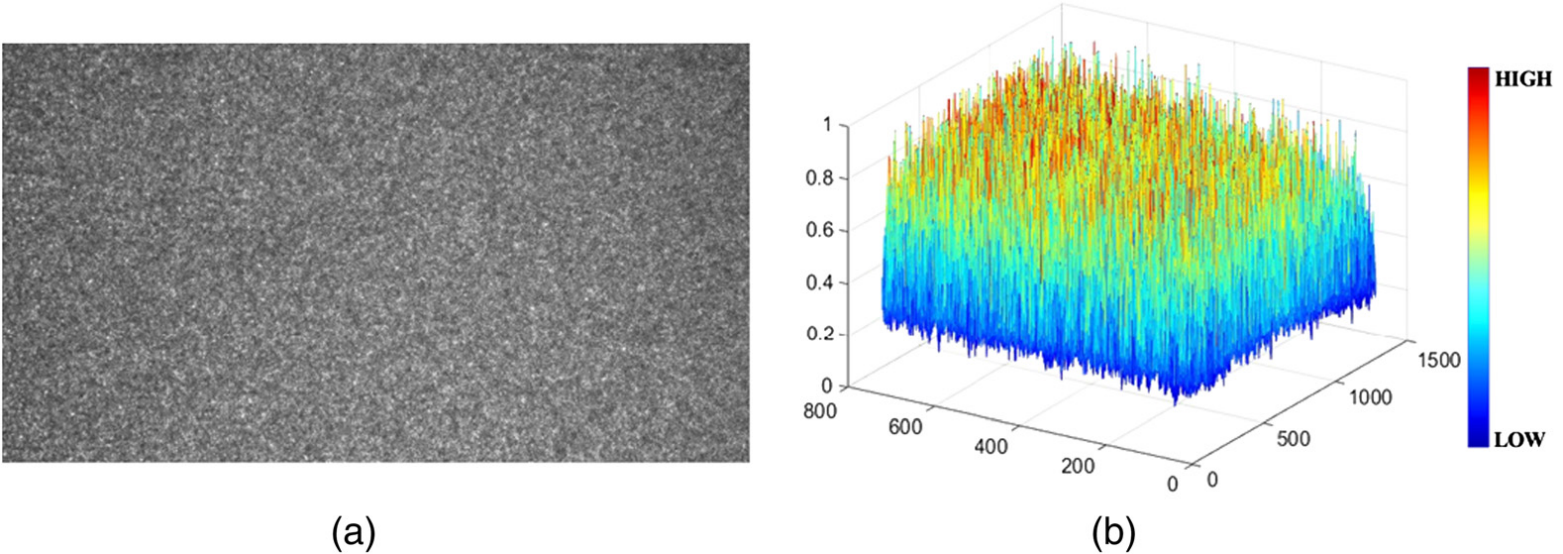
(a) 2D and (b) 3D beam distribution profiles of laser speckle image on a diffuse reflectance target with 99% reflectance.

Figures 2(a) and 2(b) show the 2D and three-dimensional (3D) beam distribution profiles of the laser speckle image on the diffuse reflectance target, respectively. The 2D beam profile was obtained by averaging the columns of Fig. 1(a). The coefficient of variation was 0.44% (19.56±0.0861), indicating a nearly even and flat laser distribution. Although the light influence of the laser projector was not measured in the LSCI, it was not observed in the LSCI because the laser projector could use an RGB laser combination to produce color projection. The LSCI was obtained at 785 nm by integrating a 785 nm diode laser and bandpass filter, avoiding potential light interference from the laser projector.

**Fig. 3:**
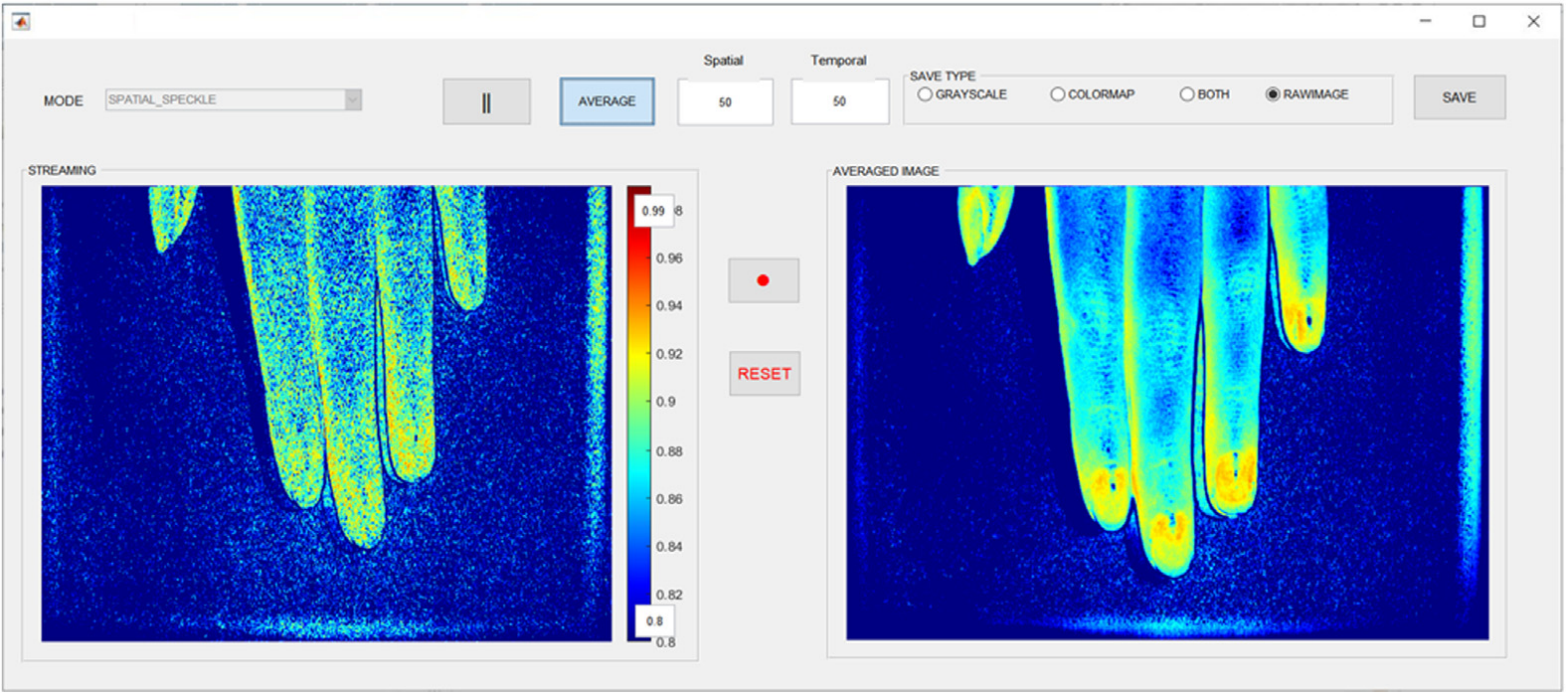
GUI software for the PM system for LSCI that captures and displays streaming laser speckle images in the “STREAMING” window and LSCIs in the “AVERAGED IMAGE” window with an adjustable pseudo-color bar.

Figure 3 shows the GUI software that captures and displays streaming laser speckle images in the “STREAMING” window and LSCIs in the “AVERAGED IMAGE” window, with an adjustable pseudo color bar. The “MODE” button enables users to switch laser speckle image and LSCI in spatial or temporal mode. The “PLAY” button starts or stops the video of the LSCI. The “AVERAGE” button averages the streaming laser speckle images and displays them in the “AVERAGED IMAGE” window. The “SAVE” button enables users to save the laser speckle image, grayscale or colormap LSCI, or both images. Video images can be acquired using the red “VIDEO RECORDING” button.

### 3.2 Evaluation of Spatial Matching in the Regions of Interest

**Fig. 4:**
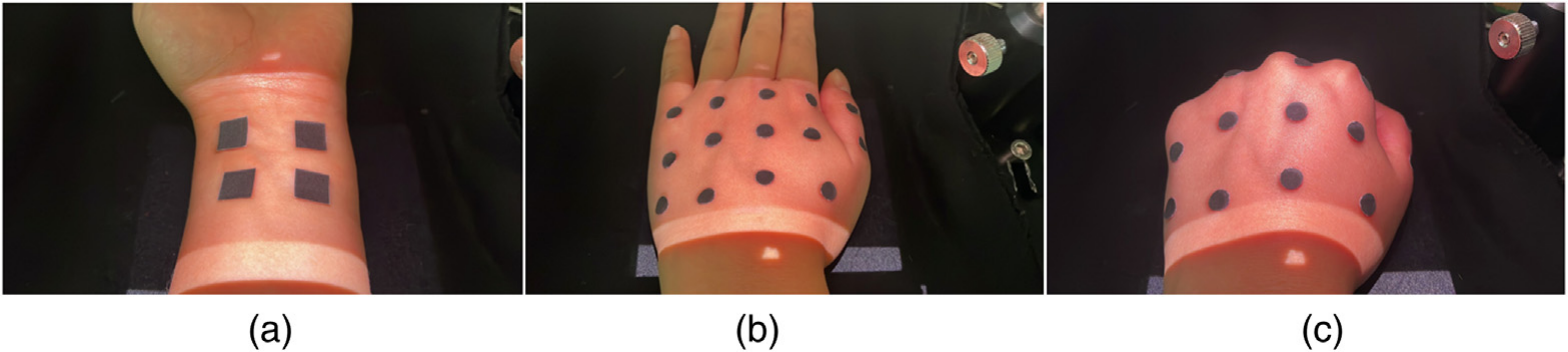
Evaluation of spatial matching in the regions of interest in which white light images were obtained and projected onto the IROI of the (a) flat volar wrist, (b) flat dorsal hand, and (c) dorsal hand fist. The square and circular markers were used to evaluate the spatial matching between the IROI and PROI.

Figures 4(a)–4(c) show the PMs of white light images on the flat volar wrist, dorsal hand with both flat and curved surfaces, and dorsal hand in a fist position to emphasize the curved surface of the human body, respectively. The SMROI was ∼2.74%, 5.56%, and 6.47% for the cases in Figs. 4(a)–4(c), respectively. Although the SMROI was not significant on the flat and curved surfaces, it was higher on the curved surface, as expected.

### 3.3 *In-Vitro* Evaluation of PMS_LSCI

**Fig. 5:**
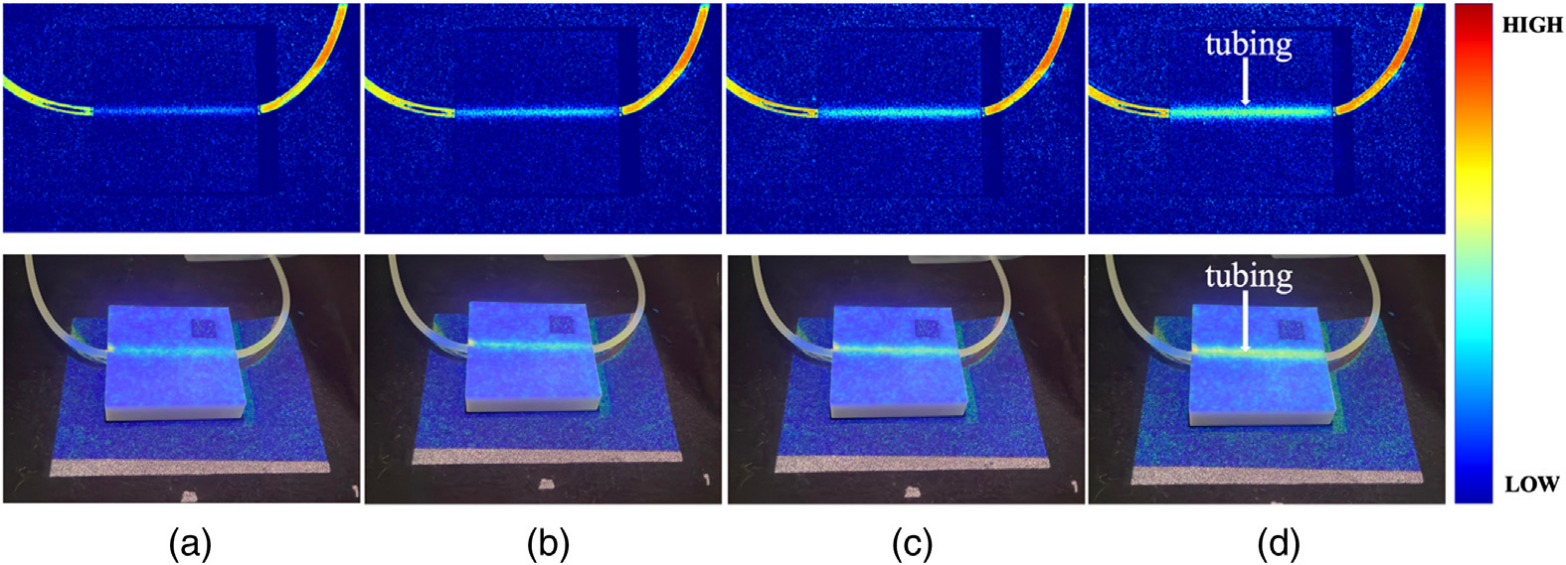
LSCIs (upper images) obtained at (a) 60, (b) 80, (c) 100, and (d) 120 RPM from vascular optical tissue phantom, and PMs (lower images) of the LSCIs on the identical vascular optical tissue phantom. The square marker was used to evaluate the spatial matching between imaging and projection regions of interest. The bright linear region is tubing with moving particles.

Figure 5 shows the LSCIs (upper images) and their PMs (lower images) on the vascular optical tissue phantom at (a) 60, (b) 80, (c) 100, and (d) 120 RPM. The pseudo color bar represents the relative velocity, where high and low velocity correspond to low (closer to speckle index 0) and high (closer to speckle index 1) laser speckle contrast, respectively. The SMROI was 1.26% at the square marker between the IROI and PROI, ensuring that the PROI was accurately matched with the IROI. As expected, the bright linear tubing region, due to the scattering of moving particles, presented velocity variations as a function of RPM. However, the outside of the tubing region presented no speckle contrast variation.

**Fig. 6:**
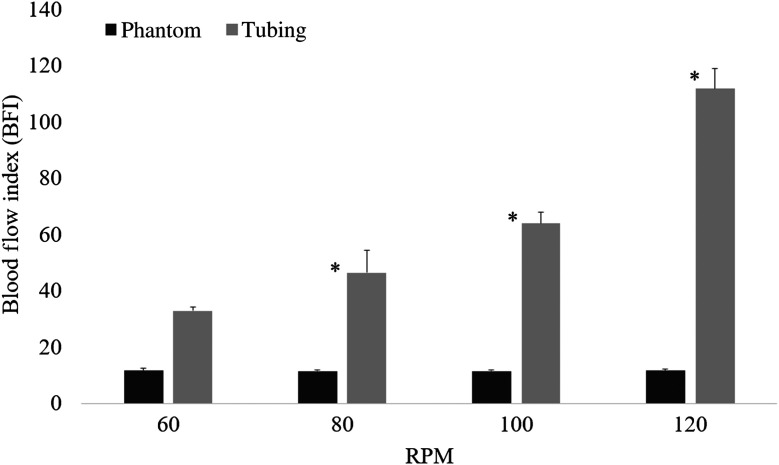
Blood flow index inside and outside the tubing region of a vascular optical tissue phantom. The tubing region presents an increase in the blood flow index as a function of RPM. The outside of the tubing region exhibits no variation in the blood flow index, as expected because there were no moving particles.

Figure 6 illustrates the blood flow indices of 32.88±1.23, 46.38±7.92, 63.83±4.02, and 111.74±7.07 at 60, 80, 100, and 120 RPM, respectively. The blood flow indices linearly increased in the tubing region as a function of RPM, and no significant differences were observed outside the tubing region.

### 3.4 *In-Vivo* Evaluation of PMS_LSCI

**Fig. 7:**
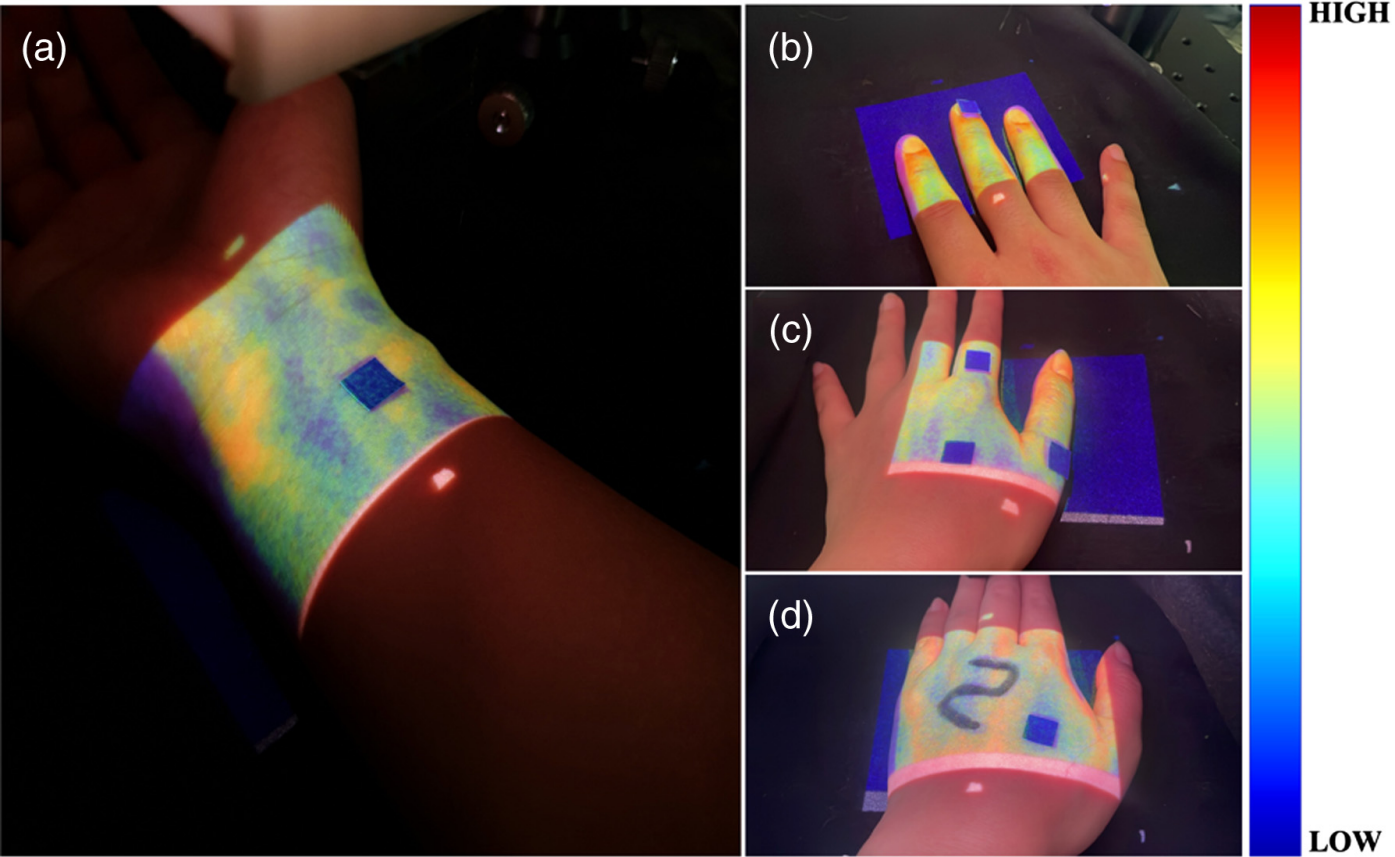
PMs of LSCIs at the (a) volar wrist, (b) fingers, (c) curvature, and (d) flat dorsal hands. The square markers and number lettering were used to evaluate the spatial matching between the IROI and PROI.

Figure 7 shows the PMs of LSCIs on the (a) flat volar wrist, (b) fingers, (c) curvature, and (d) flat dorsal hands with square markers and number lettering for the SMROI investigation. The SMROI was 2.67%, 2.75%, 4.77%, and 2.41% in Figs. 7(a)–7(d), respectively. The maximum SMROI was <5%, demonstrating high spatial matching between the IROI and PROI. Various colormaps were tested and determined as the current one in terms of visibility on skin.

**Fig. 8:**
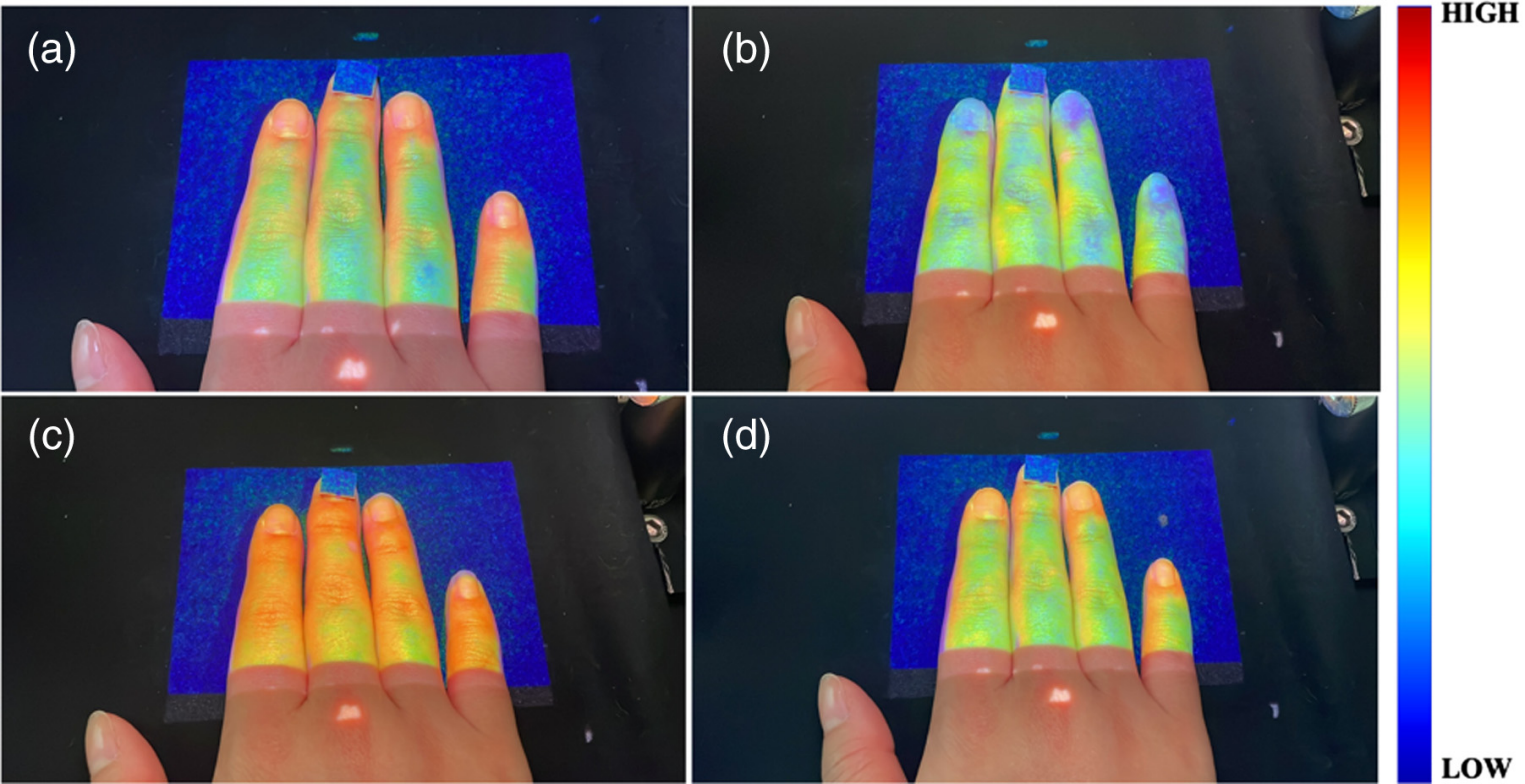
PMs of LSCIs as a function of time at the states of the (a) normal, (b) occlusion, (c) release, and (d) back to normal blood flow. The square marker was used to evaluate the spatial matching between the IROI and PROI.

Figure 8 shows the PMs of LSCIs at four different blood flows: (a) normal, (b) occlusion, (c) release, and (d) back to normal. A square marker on the middle finger was used to evaluate the SMROI. The PM provides a direct visualization of the blood flow perfusion as a function of time in the IROI. The blood flow perfusion was decreased during occlusion and increased after release. The blood flow perfusions in the normal and back to normal states were similar.

## 4 Discussion

The PM has been widely used by artists and advertisers in various settings, such as buildings, objects, the human body, media arts, and theatrical stages. Various commercial PM software packages, such as “Isadora,” “MadMapper,” “Resolume Arena,” “TouchDesigner,” “Millumin 3,” and “QLab,” have been used to project images or videos spatially onto the surfaces of objects by interfacing with a projector. In this study, PM was performed by adjusting the GUI screen to align the LSCI with the IROI. The GUI software may have the potential to enable real-time PM by employing the algorithms of commercial PM software packages and higher performance computers and projectors.

Accurate identification of lesions is important in clinical diagnosis and treatment. However, it may be challenging and time-consuming to identify the actual lesions precisely from LSCIs displayed on a monitor screen. The PM of LSCIs may address this issue by providing direct and accurate identification of the actual lesions, minimizing the spatial mismatching between the monitor screen and the actual lesion.

This study evaluated the clinical feasibility of the PMS_LSCI using a relatively low-performance computer, which may cause a time delay in real-time PM due to the computational demand of LSCI, resulting in a lower frame rate than a camera. The computation time for a single LSCI frame was ∼99.51  ms, which is comparable to 10 frames per second. Although the current GUI software did not employ the software development kit of the camera, the LSCI could be increased up to 26 frames per second by using the software development kit in a simple test program. In future studies, the GUI software should be upgraded to provide a faster frame rate by incorporating the software development kit into the algorithm. Furthermore, the image processing time of the PMS_LSCI may be enhanced for real-time PM by employing a higher performance computer equipped with an advanced graphics processing unit, which is efficient at manipulating computer graphics and image processing.

In this study, the laser source was aligned at 45 deg to the camera, resulting in the shadow artifact in the LSCI and PM, as shown in Figs. 5, 7, and 8. This artifact may be minimized by aligning the laser source and projector linearly with the camera, eliminating the need for a beam splitter. In addition, although a 90 mW laser was used in this study, a higher power laser may further improve the contrast of the raw laser speckle image and, therefore, enhance the LSCI.

The SMROI may be increased, particularly on non-planar, curved surfaces of the human body. The surface curvature artifact may be effectively resolved in future studies by using Bézier Patches’ recursive subdivision^37^ and distortion correction method^38^^–^^40^ and calibrating the 3D shapes of curved surfaces.^41^^,^^42^ In addition, the curvature artifact may be further minimized by restricting the IROI to a small region that minimizes the surface curvature artifact. In future studies, the curvature artifact may be studied using *in-vitro* objects with irregular surface curvature and applying the appropriate algorithms. In addition, the PMS_LSCI may be applied to various anatomic regions to investigate the curvature artifact in various clinical applications.

The LSCI is effective for evaluating vasculature diseases. The PMS_LSCI may provide real-time and direct monitoring of the vascular destruction on port wine stain lesions during laser therapy and, therefore, aid in determining the appropriate photon energy for each lesion based on the degree of the port wine stain. The PMS_LSCI may also be utilized in burn evaluation by providing direct and regional monitoring of the degree of burn on the lesion. In tissue transplantation, monitoring the healing progress is important to prevent tissue necrosis and determine the need for reoperation on necrotic lesions. The PMS_LSCI may also provide direct and regional monitoring of tissue necrosis on the lesion.

PM has been recently used for magnetic resonance imaging in breast-conserving surgery and cancer detection using indocyanine green fluorescence imaging.^43^^–^^45^ It may also be applied to other various 2D functional surface imaging modalities. In a previous study, actinic keratosis was successfully detected using a cross-polarization facial color imaging modality. However, it was challenging to identify and demarcate the facial actinic keratosis lesion precisely in the IROI for treatment. The PM may be combined with the imaging modality to project the processed image onto the IROI, simplifying the demarcation of actinic keratosis lesions. The PM of functional or morphological 2D surface images is expected to provide direct regional information in the IROI for diagnosis and treatment.

Although this study was focused on the PM of LSCIs to evaluate blood flow perfusion, the PMS_LSCI may also be utilized for monitoring blood flow in open cardiac and neurosurgeries that may require intensive concentration and spatial matching in the regions of interest during surgery. Previous LSCI studies have been conducted in open vasculature, providing high resolution LSCIs for fine vasculature.^19^^–^^21^^,^^46^ The detection resolution of fine vasculature may be expected to reach that in previous LSCI studies of open vasculature with an SMROI <7% based on the maximum SMROI of 6.4% on the curved surface in this study.

## 5 Conclusion

This study proposed the PMS_LSCI to observe an LSCI directly in the IROI instead of on a monitor screen by utilizing PM of the LSCI. Although further studies are needed for various clinical applications, this study demonstrated the clinical feasibility of this approach through both *in-vitro* and *in-vivo* experiments. In future studies, the computation time of image processing should be improved to achieve real-time PM of the LSCI by using higher performance computers and incorporating the software development kit of the camera into the algorithm.
